# Effect of a diet based on the dietary guidelines for americans on inflammation markers in women at risk for cardiometabolic disease: results of a randomized, controlled trial

**DOI:** 10.1186/s40795-022-00647-z

**Published:** 2022-12-27

**Authors:** Sridevi Krishnan, Tammy Freytag, Xiaowen Jiang, Gertrud U. Schuster, Leslie R. Woodhouse, Nancy L. Keim, Charles B. Stephensen

**Affiliations:** 1 Department of Nutrition, University of California Davis, Davis, CA USA; 2 Obesity and Metabolism Unit, USDA-WHNRC, Davis, CA USA; 3 Department of Pediatrics, School of Medicine, University of California San Diego, San Diego, CA USA; 4 Bioanalytical Support Laboratory, USDA-WHNRC, Davis, CA USA; 5 Immunology and Disease Prevention Unit, USDA-WHNRC, Davis, CA USA

**Keywords:** Dietary guidelines for americans, C-reactive protein, Matrix metalloproteinase-3

## Abstract

**Objective:**

To evaluate the effect of a diet pattern based on Dietary Guidelines for Americans (DGA), in a controlled feeding setting, on plasma markers of inflammation and on cytokine production by peripheral blood mononuclear cells (PBMC).

**Design:**

Women (*n =* 44) with one or more risk factors of metabolic syndrome (and BMI: 25.2-39.8 kg/m^2^) completed an 8-wk controlled feeding study. They were randomized to either a group following a diet based on DGA 2010 (DGA), or a group given a ‘typical American diet’ (TAD), based largely on a Western diet pattern. By design, women maintained their body weight. Fasting plasma and PBMC were collected at wk. 0 (baseline) and at wk. 8 (post-intervention). Sixteen plasma markers of inflammation and eight PBMC cytokines were measured at both time points, to evaluate if the diet had a significant effect on concentrations of these inflammatory markers. Data were analyzed using ANCOVA, followed by multiple-comparison adjustment using Benjamini-Hochberg method.

**Results:**

Significant changes observed in Serum Amyloid A (SAA) and Matrix Metalloproteinase 3 (MMP3) in plasma did not retain significance upon multiple comparison adjustment. SAA: *p* = 0.044, adj *p* = 0.450; DGA mean change [95% CI] = − 12.6[− 32.3 to 7.04]; TAD mean change [95% CI] = − 2.24 [− 9.99 to 5.51]. MMP3: *p* = 0.014, adj *p* = 0.35; DGA mean change [95% CI] = 2.72[− 4.16 to 9.59]; TAD mean change [95% CI] = − 0.98[− 16.7 to 14.7]). Other inflammation markers were not differently altered by DGA relative to TAD. Effect size of change (Cohens d) indicated a large/medium-large effect of intervention on MMP3 and CRP, and medium effect on IL-6.

**Conclusions:**

No statistically significant changes were observed in the immune markers examined in this study. The biological roles and magnitude of the non-significant differences seen with two variables, CRP and MMP3, suggest that they be examined in future studies.

**Trial registration:**

Clinicaltrials.gov identifier NCT02298725.

**Supplementary Information:**

The online version contains supplementary material available at 10.1186/s40795-022-00647-z.

## Introduction

For the evaluation of many health benefits, it is more useful to consider diet as a whole, as opposed to evaluating the content of individual nutrients or foods [1]. For this reason, diet patterns have been studied in association with disease and health status. Dietary patterns influence inflammation that is associated with chronic metabolic disease [2], by altering secretion of pro-inflammatory cytokines [3]. The effect of dietary patterns on inflammation has been evaluated by several researchers [4–7], mostly in the form of observational epidemiological studies but also using randomized trials. Few trials with controlled food intake have evaluated this relationship. Some of these studies have reported an inverse association between inflammation and ‘healthy’ dietary patterns or a high-quality diet [5, 8]. A reduction in inflammatory cytokines (eg: IL-6) as a result of consuming a high-quality diet has been reported by several researchers [9–12]. These studies used a Mediterranean style diet, or components of it such as olive oil or nuts to evaluate effects on inflammatory outcomes. The current study evaluated a diet based on the Dietary Guidelines for Americans (DGA) 2010. Per the Dietary Guidelines Advisory Committee, Mediterranean style diets fit the guidelines set forth by the Dietary Guidelines for a healthy diet [13]. This implies the effects between the two could be similar. However, this has not been tested in a controlled feeding trial thus far.

The DGA identifies diets that are high-quality to include more vegetables, fruits, whole grains and low-fat dairy along with reduced red-meat, added sugars and processed food high in sodium [13]. In a recently completed randomized controlled feeding trial [14], we compared a diet based on the DGA 2010 with a typical American diet (TAD), in women at risk for metabolic syndrome, based on clinical lipids, glucose and insulin profiles. Our primary outcomes, insulin and glucose tolerance remained unchanged after an 8-week exposure to these diets. We also did not observe any changes in lipids (LDLc, HDLc, total cholesterol, or triglycerides). The current report will focus on different outcomes from this study that include markers of inflammation and immune activation related to the risk of chronic inflammatory disease.

Our primary outcomes in this report include plasma markers of inflammation and immune activation, including acute phase proteins (CRP and SAA), markers of vascular inflammation (ICAM-1 and VCAM-1), pro-inflammatory cytokines (IL-1β, TNF-α and IL-6), the regulatory cytokine IL-10, chemokines (IL-8/CXCL8, IP-10/CXCL10, MCP-1/CCL2, MDC/CCL22 and eotaxin-1/CCL11) and matrix metalloproteinases (MMP) as markers of tissue remodeling (MMP-1, − 3 and − 9). In addition, cytokine production by peripheral blood mononuclear cells (PBMC) was measured following T-cell-specific stimulation (IFN-γ, IL-13, IL-17A and IL-10 as markers of Th1, Th2, Th17 and Treg cells, respectively) and treatment with bacterial lipopolysaccharide (LPS) to (primarily) stimulate monocytes (IL-1β, TNF-α, IL-6 and IL-10). Our hypothesis was that markers of inflammation, immune activation and tissue damage would decrease in the DGA group compared to the TAD group, while markers of regulatory immunity (IL-10) would increase. The DGA diet pattern has only been evaluated in a controlled feeding study twice [14, 15]. This is the first report, to our knowledge, that presents immunological outcomes following a controlled feeding intervention comparing a diet based on the DGA (with an HEI score of 98 indicating higher quality) vs a typical Western diet that is considered of relatively poor quality (HEI score: 62).

## Methods

### Study design

This report presents analysis of pre-planned outcome variables from a randomized controlled feeding trial, whose primary results have been reported previously [14]. The study enrolled women between the ages of 21-64y, BMI range between 25.2-39.8 kg/m^2^ with one or more characteristics of metabolic syndrome: impaired fasting glucose (FG > 100 and < 126 mg/dL), impaired glucose tolerance (2 h PP glucose, > 140 and < 199 mg/dL), high triglycerides (> 150 mg/dL), or low HDL-c (< 50 mg/dL). Clinical parameters from week 0 and week 8 have already been published in the primary manuscript. The study was approved by the UC Davis Institutional Review Board, and all participants signed a written informed consent form. All methods were performed in accordance with the relevant guidelines and regulations. The study is registered at clinicaltrials.gov (NCT02298725; Registered: 24/11/2014).

Detailed recruitment information, consort diagram, randomization, menu planning and intervention administration information are available in the primary manuscript (see Supplemental Fig. 1) [14] and the menu design manuscript [16]. Briefly, 44 women completed a double-blind, randomized controlled feeding intervention. Of the 44 women, 22 were randomized into the ‘DGA’ group, and would follow a diet pattern based on the DGA 2010 guidelines. The remaining 22 were randomized to follow a typical American diet (TAD). By design, the energy content of both diets were meant to maintain body weight, as a means to evaluate the effect of the pattern independent of body weight change, on several health outcomes. Energy requirements were calculated by measuring resting metabolic rate using metabolic carts (TrueOne 2400, Parvomedic), with adjustments for physical activity energy expenditure (measured using activity monitors (Actical, Phillips Respironics), and estimated thermic effect of food. Body weight was monitored twice weekly to ensure steady state. As part of the intervention, all food and beverages were provided to the participants for a duration of 8 weeks. Participants were advised to consume all foods and beverages given to them, and not to consume anything outside of what was provided to them. Dietary adherence to the intervention diets was tracked using several methods including (a) a daily checklist that participants filled out with when and how much of given food was consumed by meal, and document what food outside of the study diet they consumed (b) weigh backs of containers that participants brought back twice a week to the study center (and were advised to not clean out or wash containers at home), (c) urinary nitrogen “recovery” estimated as a ratio of dietary nitrogen intake and excretion based on three 24 h urine collected during the 8-week study. Adherence was between 80 and 98% for all provided foods and beverages in both groups [16]. Several aspects of the participants’ health were evaluated at baseline (wk0), following 2 weeks of exposure to the assigned diet (wk2) and at the end of the 8-week exposure (wk8), as reported earlier. The outcomes of interest in the current manuscript are plasma and PBMC-derived cytokines and other markers of inflammation or immune activation. These were measured at wk0 and wk8. On days that these measures were evaluated, participants arrived at the study center following an overnight fast. Fasting blood was collected, processed, aliquoted, and stored at − 80 °C.

### Plasma cytokine parameters

Electrochemiluminescence (ECL) technology from Meso Scale Discovery (MSD; Rockville, MD) was used following the manufacturer’s instructions. Human Proinflammatory V-Plex kits were used to determine the duplicate concentrations of IL-1β, IL-6, IL-8, IL-10, and TNF-α in EDTA plasma. Vplex Chemokine Panel 1 kits were used to measure Eotaxin, IP-10, MCP-1 and MDC in EDTA plasma. Matrix metalloproteinases MMP-1, MMP-3, MMP-9 were measured in human heparin plasma samples using MSD Ultrasensitive kits. Data were obtained with the SECTOR Imager 2400. V-plex kits are consistent between lots and include low, medium, and high controls. Analytical validation is performed on kits from each lot, measuring sensitivity, accuracy, precision, specificity, and sample values. MSD technology offers high sensitivity and broad dynamic range and provides highly reproducible results.

### PBMC isolation and culture for cytokine production

Briefly, blood for PBMC isolation was collected in sodium-heparin tubes and processed within 1 hour after the blood draw. Blood was centrifuged at 800 x g for 10 min at 20 °C. After the plasma was removed, the buffy coat was transferred to a new 15 mL conical tube and diluted with HBSS (Hank’s Balanced Salt Solution; Invitrogen, Carlsbad, CA) to a volume of 7 mL. PBMC were isolated using a standard density step gradient (using Histopaque 1077; Sigma, St. Louis, MO). Cells of the PBMC layer were washed with ice-cold HBSS, suspended in 1 mL cold sterile phosphate buffered saline (PBS) and counted. The cells were diluted in complete Russ-10 medium (Russ-10 prepared as described [17] plus 10% fetal bovine serum), plated at the density of 1 × 10^6^ cells/mL in a 96-well plate (200 μL per well) and cultured at 37^o^ C in a 5% CO_2_ atmosphere. Supernatants were collected after 24 h (LPS treatment) or 48 h (ant-CD3/CD28 treatment) and stored at − 80 °C until used for further analyses. PBMC cultures were stimulated with final concentration of 5 ng/mL lipopolysaccharide (LPS) (List Biological, Campbell, CA; from *E. coli* 0111:B4 and dissolved in endotoxin-free water) in complete Russ-10 medium; endotoxin free water was used as a negative control. For T-cell stimulation (anti-CD3/CD28) the wells were precoated with combined anti-human CD3 3 μg/mL (isotype IgG2a; eBioscience, cat# 16-0037) and anti-human CD28 3 g/ml (isotype IgG1; eBioscience, San Diego, CA; cat# 16-0289) or as negative control with mouse igG2a Isotype Control (eBioscience, cat# 16-0289) plus mouse IgG1 isotope Control (eBioscience, cat# 16-4714).

### Measuring of cytokines in supernatants from cultured PBMC

PBMC supernatants from cultures stimulated with anti-CD3/CD28 antibodies, or isotype control antibodies, were tested for IL-2, IL-10, IL-13, IL-17A and INF-**γ**. Supernatants from LPS-stimulated cultures, or negative control cultures, were tested for IL-1β, IL-6, IL-10 and TNF-α. Cytokines were assayed using electrochemiluminescence based detection platform with multiplexed immunoassays using the U-PLEX system from MSD (Mesoscale Discovery, LLC, Rockville, Maryland, USA) as previously described [18].

### Statistical analysis

All variables were evaluated for normality using Shapiro Wilk tests, and were transformed to achieve normality, based on their w-score, and qq plots. Huber and Cauchy tests were used to determine if there were outliers, transformed data did not have any outliers. The primary question was to evaluate if there was a significantly different effect of the diet treatments on plasma and PBMC based cytokines. Transformed data were used in an analysis of covariance evaluating group differences, with week 8 data as the outcome variable, adjusted for baseline data (wk 0) as the covariate. BMI category, menopausal status and body weight were used as covariates in order to verify if these were affecting outcomes. *P*-values for treatment effects were adjusted for multiple comparison effects using Benjamini-Hochberg tests. 95% confidence interval of change (wk8-wk0) were calculated and presented. F-tests were used to determine if the variance between the change (wk8-wk0) in groups were equal. Clinical cut-off values for various inflammatory cytokines from previously published literature were used to classify study participants at baseline. Contingency tables and chi square test for difference in proportions were used to determine whether these clinical cut-off categories were different between groups at baseline, and following the intervention. All analyses were done using R statistical software (RStudio version 1.2.1555) [19] and graphs were made in JMP Pro 14.1 (SAS Institute, Gary NC).

## Results

Study participants were women between 21 and 64 y of age, with body mass index (BMI) ranging from 25.2 to 39.8 kg/m^2^ (see Supplemental Table 1 for further details). Age, BMI and other physical and clinical characteristics (including fasting glucose, insulin, lipids, systolic and diastolic blood pressure, and indexes related to glucose homeostasis and insulin resistance/sensitivity) did not differ between the dietary intervention groups at baseline, as previously reported [14].

**Fig. 1:**
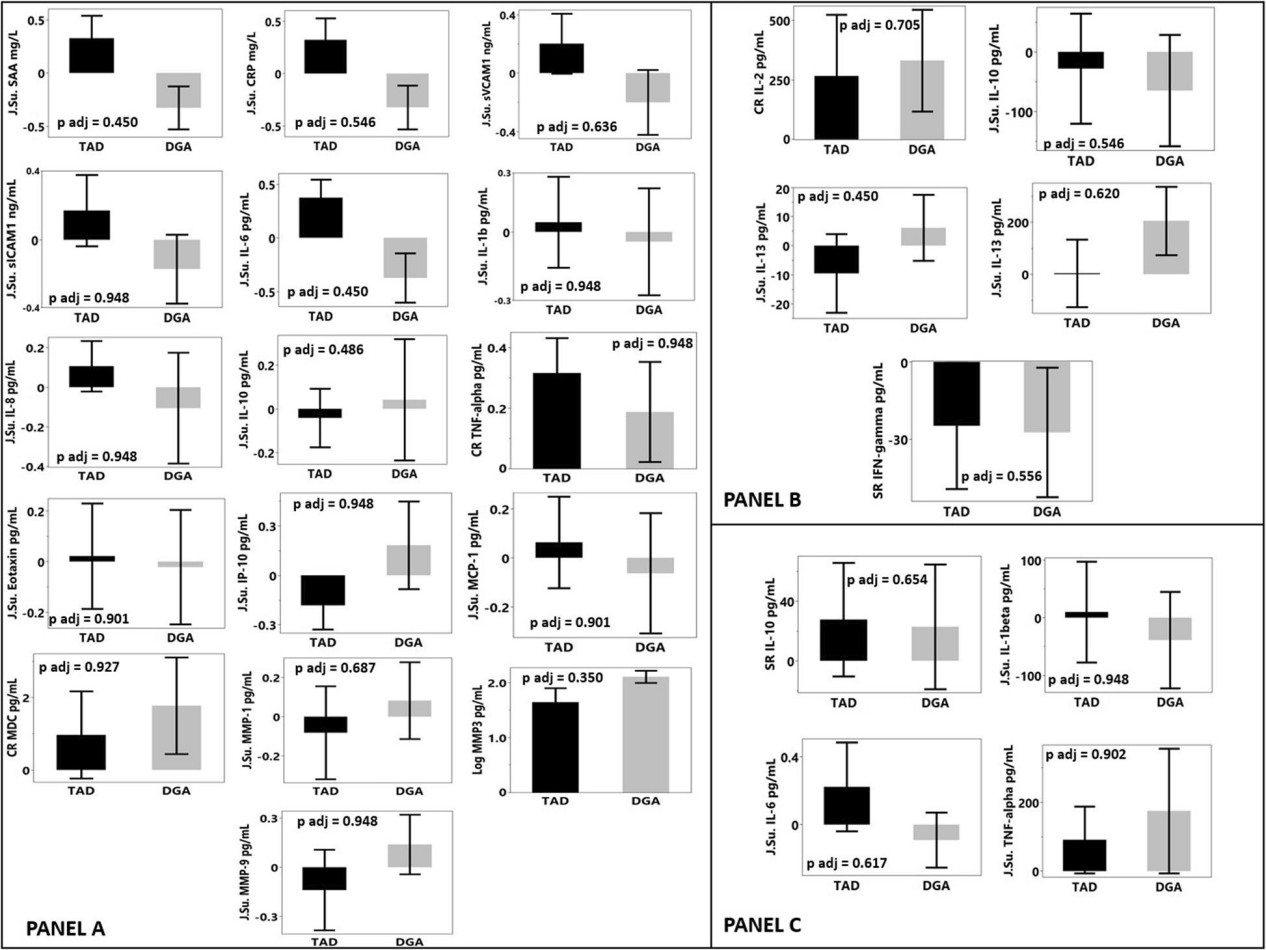
**A** Difference between Wk8 and Wk0 for all fasting cytokine parameters measured in plasma and derived from PBMCs with p-values and Benjamini-Hochberg adjusted *p*-values inset. One-way ANCOVA with adjustment for baseline values indicated no significant differences between DGA and TAD groups. Values are mean ± SEM. J-Su – Johnson normalizing, unbounded (Su), J. Sb – Johnson normalizing bounded (Sb) Log – logarithmic transformation, CR – cube root transformation; **B** Difference between Wk8 and Wk0 for all fasting cytokine parameters measured in PBMCs stimulated with anti-CD3 and anti-CD28 antibody, with *p*-values and Benjamini-Hochberg adjusted p-values inset. One-way ANCOVA with adjustment for baseline values indicated no significant differences between DGA and TAD groups. Values are mean ± SEM. J. Su – Johnson normalizing unbounded (Su), SR – square root transformation, CR – cube root transformation; **C** Difference between Wk8 and Wk0 for all fasting cytokine parameters measured in PBMCs stimulated with LPS with p-values and Benjamini-Hochberg adjusted p-values inset. One-way ANCOVA with adjustment for baseline values indicated no significant differences between DGA and TAD groups. Vales are mean ± SEM. J. Su – Johnson normalizing unbounded (Su), SR – square root transformation

A summary of all week 0 and week 8 immunological measures in plasma and PBMC derived cytokines induced with LPS and anti-CD3/CD28 are presented in Table 1. It is important to note that BMI, body weight or menopausal status were not significant covariates for any of the parameters. Hence, the numbers reported here reflect unadjusted model outcomes (i.e., no covariate). The change in plasma cytokines between week 8 and week 0 for DGA and TAD groups are presented in Fig. 1 Panel A. The change mean and 95% confidence intervals for DGA and TAD groups are presented in Table 2. F-tests indicated unequal variance between groups in SAA (*p* < 0.001), CRP (p < 0.001), IL-6 (*p* = 0.004), IL-8 (p < 0.001), IL-10 (p < 0.001), TNF-a (p < 0.001), IP-10 (p < 0.001), MMP-1 (*p* = 0.038) and MMP-9 (p < 0.001). The significantly greater reduction in plasma SAA following the 8-week exposure to the DGA diet, compared to the women on the TAD diet (*p* = 0.044), became non-significant with multiple comparison correction (*p* = 0.45). Similarly, the statistically significant increase in plasma MMP-3 in the DGA group compared to a reduction following the TAD intervention (*p* = 0.014), also became non-significant with multiple comparison correction (*p* = 0.35). Although plasma IL-10 tended to increase in DGA compared to TAD, this was not significant (*p = 0.097*). Conversely, plasma IL-6 decreased following the DGA intervention and increased in the TAD intervention group, but this difference did not reach statistical significance (*p = 0.072*). Once multiple comparison adjustments were performed group mean differences between IL-10 or IL-6 were not significant.

**Table 1:** Immunological parameters measured at fasting in plasma and PBMCs at weeks 0 and 8. Values are mean ± SEM

Parameter	TAD						DGA					
	Week 0			Week 8			Week 0			Week 8		
***Serum***												
**TNF-α (pg/mL)**	1.99	±	0.127	2.20	±	0.150	2.23	±	0.213	2.23	±	0.214
**IL-10 (pg/mL)**	0.283	±	0.0227	0.286	±	0.0181	0.529	±	0.146	0.840	±	0.441
**IL-1β (pg/mL)**	0.121	±	0.0128	0.125	±	0.0176	0.122	±	0.009	0.125	±	0.008
**IL-6 (pg/mL)**	1.46	±	0.293	1.90	±	0.403	1.45	±	0.289	1.35	±	0.255
**IL-8 (pg/mL)**	4.02	±	0.339	4.57	±	0.335	6.22	±	0.677	7.19	±	1.23
**IP-10 (pg/mL)**	579	±	135	569	±	116	698	±	165	1450	±	593
**Eotaxin (pg/mL)**	124	±	14.7	160	±	34.3	155	±	20.8	143	±	11.4
**MCP-1 (pg/mL)**	105	±	9.21	113	±	12.4	112	±	6.84	115	±	8.83
**MDC (pg/mL)**	962	±	80.6	1045	±	107	901	±	71.8	981	±	60.3
**MMP-1 (pg/mL)**	2.47	±	0.392	2.28	±	0.313	2.49	±	0.509	2.50	±	0.408
**MMP-3 (pg/mL)**	9.60	±	0.875	8.80	±	0.815	9.70	±	1.03	11.1	±	1.13
**MMP-9 (pg/mL)**	44.1	±	6.70	45.2	±	9.48	31.2	±	3.2	33.9	±	4.03
**CRP (mg/L)**	8.05	±	1.53	9.37	±	1.60	10.6	±	2.85	6.90	±	1.18
**SAA (mg/L)**	13.9	±	4.89	11.0	±	2.43	22.3	±	9.9	9.69	±	2.08
**sICAM1 (ng/mL)**	552	±	40.5	565	±	29.3	611	±	41.2	593	±	48.4
**sVCAM1 (ng/mL)**	551	±	33.8	574	±	29.8	644	±	34.2	632	±	37.5
***PBMCs***												
**CD3/28-IL-10 (pg/mL)**	313	±	59.2	285	±	77.8	504	±	67.8	439	±	76.4
**CD3/28-IL-13 (pg/mL)**	62.3	±	11.2	52.8	±	10.7	68.9	±	8.88	75.0	±	7.52
**CD3/28-IL-17A (pg/mL)**	610	±	133	613	±	140	651	±	101	856	±	137
**CD3/28-IL-2 (pg/mL)**	651	±	140	917	±	289	689	±	130	1020.0	±	187
**CD3/28-IFNγ (pg/mL)**	111	±	22.5	86.1	±	18.0	156	±	21.9	129	±	18.6
**LPS-IL-10 (pg/mL)**	103	±	34.5	131	±	28.6	124	±	24.6	147	±	34.9
**LPS-IL-1b (pg/mL)**	438	±	129	329	±	108	370	±	92.5	280	±	76.4
**LPS-IL-6 (pg/mL)**	0.480	±	0.247	0.571	±	0.305	0.468	±	0.312	0.313	±	0.153
**LPS-TNF- α (pg/mL)**	361	±	91.2	353	±	93.2	317	±	57.5	448	±	166

**Table 2:** Clinical cut-off-based classification of participants, at baseline and post-intervention. Chi square tests for difference of proportions indicated no difference between groups at week 0 or 8

Cytokine parameter	Week 0		Week 0		Week 8	
	All (n)	All (%)	TAD	DGA	TAD	DGA
**CRP (High: > 3 mg/L)** [20]						
High	32	72.7	17	15	16	14
Normal	12	27.3	5	7	6	8
**CRP (High: > 2 mg/L)** [20]						
High	36	81.8	19	17	18	15
Normal	8	18.2	3	5	4	7
**CRP (High: > 1 mg/L)** [20]						
High	41	93.2	21	20	22	20
Normal	3	6.8	1	2	0	2
**SAA (High:> 40 mg/L)** [21]						
High	4	9.1	2	2	1	0
Normal	40	90.9	20	20	21	22
**SAA (High:> 20 mg/L)** [21]						
High	8	18.2	2	6	2	2
Normal	36	81.8	20	16	20	20
**sICAM1 (High: >22μg/mL)** [22]						
High	0	0.0	0	0	0	0
Normal	44	100.0	22	22	22	22
**sVCAM1 (High: >1150μg/mL)** [22]						
High	0	0.0	0	0	0	0
Normal	100	100.0	22	22	22	22
**IL-1β (High: > 12 ng/L)** [23]						
High	0	0.0	0	0	0	0
Normal	44	100.0	22	22	22	22
**IL-6 (High: > 5 ng/L)** [23]						
High	0	0.0	0	0	0	0
Normal	44	100.0	22	22	22	22
**IL-8 (High: > 63 ng/L)** [24]						
High	0	0.0	0	0	0	0
Normal	44	100.0	22	22	22	22
**TNF-α (High: > 3 ng/L)** [25]						
High	5	11.4	0	5	2	5
Normal	39	88.6	22	17	20	17

The change in anti-CD3/CD28 treated PBMC-derived cytokines between week 0 and week 8 for both groups are presented in Fig. 1 Panel B. F-tests suggested no difference in variance between groups in these outcomes. The change in LPS treated PBMC-derived cytokines between week 8 and week 0 are presented in Fig. 1 Panel C. F-tests suggested unequal variances in change between DGA and TAD groups in IL-6 (*p* = 0.033) and TNF-a (*p* = 0.005). No significant differences were identified in the mean change between groups.

**Fig. 2:**
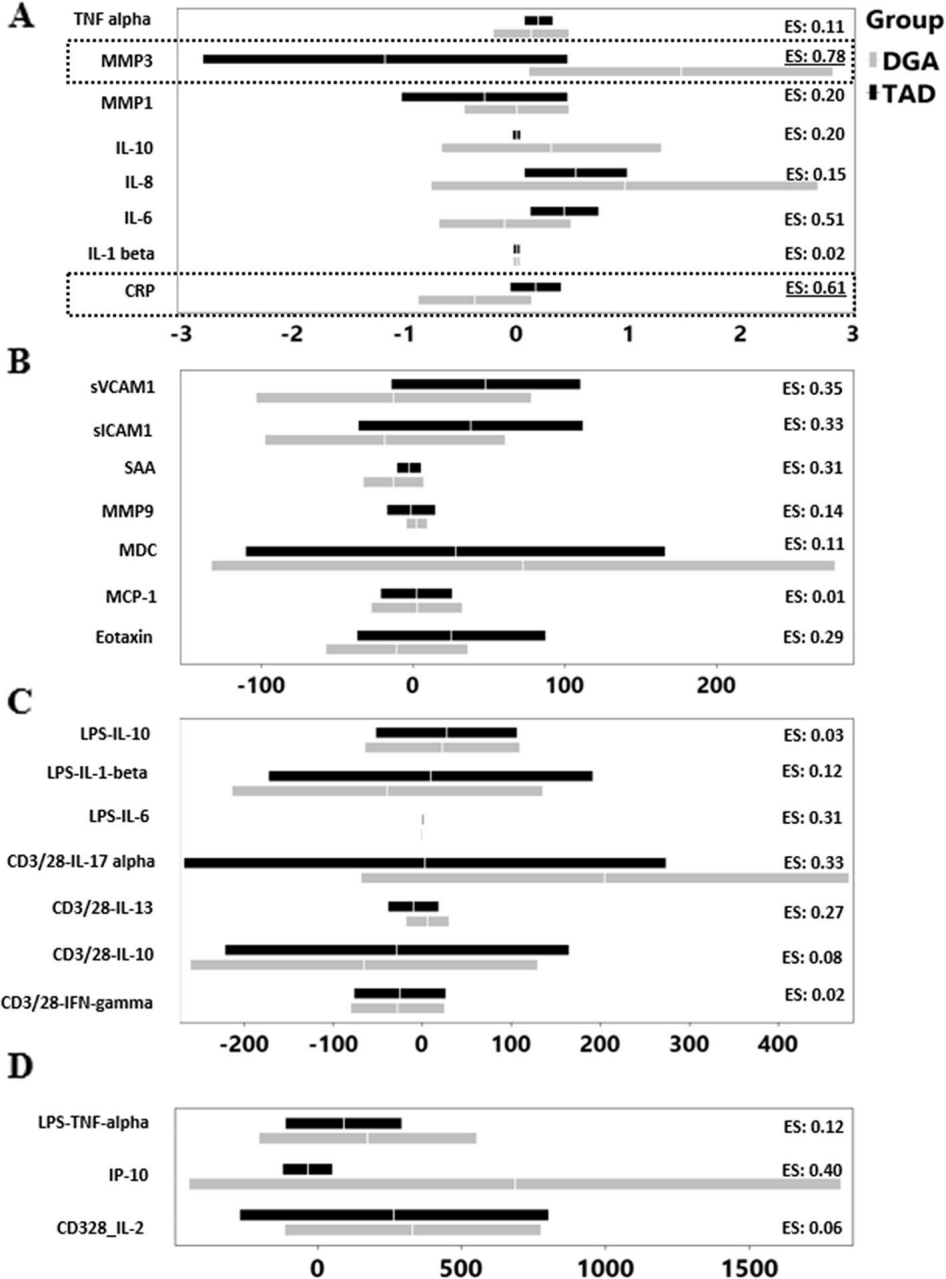
The lower and upper CI and mean change between wk8-wk0 in TAD (black bars) and DGA (grey bars), inset with effect size of change (ES) calculated as mean /pooled standard deviation on the right corner are shown in panels **A** – **D**. Panels **A** and **B** show data from fasting plasma, while **C** and **D** show data from PBMC’s, with the exception of IP-10 in panel **D** (which was graphed with other variables that could share the x-axis). The upper and lower CI are depicted at the ends of the bars, and the mean is represented by the while line/space at the center of each bar. Large intervals were noticed in several immunological parameters, suggesting low power. Based on effect size of change observed, both MMP3 and CRP have medium to large effect sizes (denoted by black dotted rectangles), trailed by IL-6 and IP-10. LPS-IL-6 also had slightly more than low effect size (at 0.31). While MMP3 and IP-10 increased following DGA, and decreased following TAD, and the reverse was true in CRP and IL-6

Confidence intervals and effect size (Cohen’s d [26]) of change between wk8 and wk0 for DGA and TAD groups are presented in Fig. 2 (actual numbers in Supplemental Table 2). While no significant differences were identified, MMP3 had the largest effect size, followed by CRP in the medium to large range. This was followed by IL-6 and IP-10 with medium or slightly larger than small effect sizes. CRP and IL-6 decreased following DGA, and increased following TAD, while MMP3 and IP-10 both increased following the DGA intervention, relative to TAD.

In the present study, between 72.7 to 93.2% of volunteers, had “elevated” CRP at baseline depending on the cutoff (Table 2). The percent of volunteers with ‘elevated’ values at baseline ranged from 0 to 11.4% based on cutoffs for SAA, ICAM-1, VCAM-1, IL-1β, IL-6, IL-8 and TNF-α. No significant differences were seen in the proportion of volunteers that had ‘elevated’ and ‘normal’ values based on these cut-offs between groups.

## Discussion

In a group of 44 overweight/obese women with one or more characteristics of metabolic syndrome, following a diet based on the DGA 2010 for 8 weeks resulted in magnitudinal changes in SAA, MMP3, s-ICAM1, s-VCAM1, MDC, Eotaxin, LPS stimulated TNFα, CD3/CD28 treated IL-17β compared to the group following a ‘typical American diet’. Statistically significant changes in SAA and MMP3 did not remain significant after multiple comparison correction, however, the study was powered to measure primary outcomes that were previously published [14]. Immunologic variables often vary substantially among individuals, and the study would likely have had a larger sample size had it been powered to find differences in SAA or CRP for instance. This was also evident from the large effect size of change observed in MMP3, and medium effect sizes in CRP and IL-6. Hence, these magnitudinal changes, albeit not statistically significant will be discussed here to benefit future studies.

The Mediterranean diet, one type of Dietary Guidelines recommended diet [27], has been shown to improve immunological biomarkers including CRP, IL-6 and monocyte adhesion molecules in Mediterranean [28] and US cohorts [29]. Similarly, the UK-dietary guidelines (which have overlaps with the Mediterranean diet pattern) were compared to a typical UK diet (not a controlled feeding study, but with partial provision of select foods) over a 12-week intervention, the UK dietary guidelines diet also reduced plasma CRP [30].

However, several of the cytokines that were measured in this study did not respond differently to the two diet patterns. Data from the FAVRIT study [31], which involved an 8-week intervention of increased fruits and vegetables, without change in body weight, identified reduced SAA, but no change in CRP or other inflammatory cytokines. They suspected that an 8-week duration may not be adequate to impart effects of a higher fruit and vegetable diet on inflammatory cytokines. This may be the case in the present study as well. The circulating CRP distribution in this study is on the higher end of the spectrum when compared to what is reported from other population-based studies [32]. A close look at the 95% CI in the current study suggests change in opposite directions in CRP following the intervention. In the Dallas Heart Study, 51 and 58% of White and Black women respectively were reported to have > 3 mg/L [33]. CRP > 10 mg/L was reported in less than 5% of the population in a large cohort (*n =* 22,403) of men and women from the Women’s Health Study, Women’s Health Initiative, Air Force/Texas Coronary Atherosclerosis Prevention Study and the Physician’s Health Study [32]. In the current study, using a > 3 mg/L cutoff, 72.7% of women fell into a “high” inflammatory category, and 29.5% using a > 10 mg/L cutoff. This would be expected since these women had one or more characteristics of metabolic syndrome. Despite this, diet did not have an impact on CRP concentrations.

Matrix metalloproteinases are a family of extracellular matrix remodeling proteases that are zinc and calcium-dependent [34]. There are several groups of metalloproteinases, of which MMP-1 is a soluble collagenase, MMP-3 a stromelysin, and MMP-9 a gelatinase [35]. Of particular interest in this report, is MMP3, which was increased following the 8-week DGA diet, compared to the TAD group. Often called the anti-adipogenic factor, in in vitro systems, this stromelysin has been reported to inhibit hyperplastic adipose expansion [36], and is expressed primarily in pre-adipocytes [37]. Knocking out the MMP3 gene results in adipocyte hypertrophy in mice when exposed to a high fat diet, as well as hyperphagia and obesity [38]. In C57BL/6 J mice, Wu et al. [39] reported that MMP3 expression was downregulated, in order for high fat diet to induce adipogenesis, resulting in hypertrophy and obesity. These data suggest that increase in MMP3 observed following DGA diet may impart anti-adipogenic properties, independent of weight change. Specific mechanistic information of which nutrients and how they influence MMPs is not very well understood [34]. However, effect of DGA being anti-adipogenic is in line with the DGA being considered a high quality and healthier diet, compared to TAD.

## Conclusions and limitations

This study identified only a marginal difference between the DGA and TAD diet interventions, this could be due to several reasons. The duration of the study was short, and it is likely that a 12- or 16- week exposure to these diet patterns may have revealed stronger effects, as well as in more outcome variables. The sample size was also small, and was powered for primary outcome variables (insulin and glucose) and not for immune parameters. Including several secondary immune endpoints also necessitated the use of multiple comparison corrections, further reducing the ability to detect statistically significant changes. The study participants were both pre-and postmenopausal women. However, controlled feeding trials are challenging to implement and complete, and the DGA has never been tested for its effect on inflammatory markers in a controlled feeding setting before the present study. In summary, a diet based on the DGA 2010 was able to induce some changes that are anti-inflammatory, but this will need to be verified in larger trials with longer durations.

## Supplementary Information

**Additional file 1.**

## Data Availability

The datasets used and/or analyzed during the current study is a component of a larger study, where investigators have yet to publish all outcomes entirely. Hence, data will be posted together at a later date. In the meantime, the data for this report are available from the corresponding author on reasonable request.

## Abbreviations

IL-6: interleukin-6
IL-10: interleukin-10
MMP1, 3 and 9: .matrix metalloproteinase-, -3 and -9
CRP: C-reactive protein.
DGA: Dietary Guidelines for Americans
TAD: Typical American Diet

## References

[1] Kong LC, Holmes BA, Cotillard A, Habi-Rachedi F, Brazeilles R, Gougis S (2014). Dietary patterns differently associate with inflammation and gut microbiota in overweight and obese subjects. PLoS One.

[2] Schulze MB, Hoffmann K, Manson JE, Willett WC, Meigs JB, Weikert C (2005). Dietary pattern, inflammation, and incidence of type 2 diabetes in women. Am J Clin Nutr.

[3] Silveira BKS, Oliveira TMS, Andrade PA, Hermsdorff HHM, Rosa COB, Franceschini SDCC (2018). Dietary Pattern and Macronutrients Profile on the Variation of Inflammatory Biomarkers: Scientific Update. Cardiol Res Pract.

[4] Drewnowski A, Fiddler EC, Dauchet L, Galan P, Hercberg S (2009). Diet quality measures and cardiovascular risk factors in France: applying the Healthy Eating Index to the SU.VI.MAX study. J Am Coll Nutr.

[5] Meyer J, Döring A, Herder C, Roden M, Koenig W, Thorand B (2011). Dietary patterns, subclinical inflammation, incident coronary heart disease and mortality in middle-aged men from the MONICA/KORA Augsburg cohort study. Eur J Clin Nutr.

[6] Nettleton JA, Steffen LM, Schulze MB, Jenny NS, Barr RG, Bertoni AG (2007). Associations between markers of subclinical atherosclerosis and dietary patterns derived by principal components analysis and reduced rank regression in the Multi-Ethnic Study of Atherosclerosis (MESA). Am J Clin Nutr.

[7] Cowan SF, Leeming ER, Sinclair A, Dordevic AL, Truby H, Gibson SJ (2020). Effect of whole foods and dietary patterns on markers of subclinical inflammation in weight-stable overweight and obese adults: a systematic review. Nutr Rev.

[8] Esposito K, Marfella R, Ciotola M, Di Palo C, Giugliano F, Giugliano G (2004). Effect of a mediterranean-style diet on endothelial dysfunction and markers of vascular inflammation in the metabolic syndrome: a randomized trial. JAMA.

[9] Chrysohoou C, Panagiotakos DB, Pitsavos C, Das UN, Stefanadis C (2004). Adherence to the Mediterranean diet attenuates inflammation and coagulation process in healthy adults: The ATTICA Study. J Am Coll Cardiol.

[10] Urpi-Sarda M, Casas R, Chiva-Blanch G, Romero-Mamani ES, Valderas-Martínez P, Arranz S (2012). Virgin olive oil and nuts as key foods of the Mediterranean diet effects on inflammatory biomakers related to atherosclerosis. Pharmacol Res.

[11] Smidowicz A, Regula J (2015). Effect of nutritional status and dietary patterns on human serum C-reactive protein and interleukin-6 concentrations. Adv Nutr.

[12] Mena MP, Sacanella E, Vazquez-Agell M, Morales M, Fitó M, Escoda R (2009). Inhibition of circulating immune cell activation: a molecular antiinflammatory effect of the Mediterranean diet. Am J Clin Nutr.

[13] United States Department of Agriculture: Dietary Guidelines for Americans 2010. In: *Dietary Guidelines for Americans.* Edited by Services United States Department of Health and Human Servies, 7th Edition edn. Washington DC: US Government Printing Office; 2010. 10.3945/an.111.000430 PMC3090168 22332062

[14] Krishnan S, Adams SH, Allen LH, Laugero KD, Newman JW, Stephensen CB (2018). A randomized controlled-feeding trial based on the Dietary Guidelines for Americans on cardiometabolic health indexes. Am J Clin Nutr.

[15] Schroeder N, Park YH, Kang MS, Kim Y, Ha GK, Kim HR (2015). A randomized trial on the effects of 2010 Dietary Guidelines for Americans and Korean diet patterns on cardiovascular risk factors in overweight and obese adults. J Acad Nutr Diet.

[16] Krishnan S, Lee F, Burnett DJ, Kan A, Bonnel EL, Allen LH, et al. Challenges in Designing and Delivering Diets, and Assessing Adherence: A Randomized Controlled Trial Evaluating the 2010 Dietary Guidelines for Americans. *Current Developments in Nutrition*. 2020; In Press. 10.1093/cdn/nzaa022 PMC7066378 32190808

[17] Stephensen CB, Rasooly R, Jiang X, Ceddia MA, Weaver CT, Chandraratna RA (2002). Vitamin A enhances in vitro Th2 development via retinoid X receptor pathway. J Immunol.

[18] Kewcharoenwong C, Schuster GU, Wessells KR, Hinnouho GM, Barffour MA, Kounnavong S, et al. Daily Preventive Zinc Supplementation Decreases Lymphocyte and Eosinophil Concentrations in Rural Laotian Children from Communities with a High Prevalence of Zinc Deficiency: Results of a Randomized Controlled Trial. J Nutr. 2020. 10.1093/jn/nxaa037 32119742

[19] R Core Team: A language and environment for statistical computing. Vienna; 2013.

[20] Hwang YG, et al. Differential response of serum amyloid A to different therapies in early rheumatoid arthritis and its potential value as a disease activity biomarker. Arthritis Res Ther. 2016;18:108. 10.1186/s13075-016-1009-y. 10.1186/s13075-016-1009-y PMC4869396 27188329

[21] Pearson TA, Mensah GA, Alexander RW, Anderson JL, Cannon RO, Criqui M (2003). Markers of inflammation and cardiovascular disease: application to clinical and public health practice: A statement for healthcare professionals from the Centers for Disease Control and Prevention and the American Heart Association. Circulation..

[22] Andrýs C, Pozler O, Krejsek J, Derner V, Drahosová M, Kopecký O (2000). Serum soluble adhesion molecules (sICAM-1, sVCAM-1 and sE-selectin) in healthy school aged children and adults. Acta Med (Hradec Kralove).

[23] O'Neill CM, Lu C, Corbin KL, Sharma PR, Dula SB, Carter JD (2013). Circulating levels of IL-1B+IL-6 cause ER stress and dysfunction in islets from prediabetic male mice. Endocrinology..

[24] Zhang J, Bai C (2017). Elevated Serum Interleukin-8 Level as a Preferable Biomarker for Identifying Uncontrolled Asthma and Glucocorticosteroid Responsiveness. Tanaffos..

[25] Todd J, Simpson P, Estis J, Torres V, Wub AH (2013). Reference range and short- and long-term biological variation of interleukin (IL)-6, IL-17A and tissue necrosis factor-alpha using high sensitivity assays. Cytokine..

[26] Lakens D (2013). Calculating and reporting effect sizes to facilitate cumulative science: a practical primer for t-tests and ANOVAs. Front Psychol.

[27] Nadeem N, Woodside JV, Neville CE, McCall DO, McCance D, Edgar D (2014). Serum amyloid A-related inflammation is lowered by increased fruit and vegetable intake, while high-sensitive C-reactive protein, IL-6 and E-selectin remain unresponsive. Br J Nutr.

[28] Rifai N, Ridker PM (2003). Population distributions of C-reactive protein in apparently healthy men and women in the United States: implication for clinical interpretation. Clin Chem.

[29] Khera A, McGuire DK, Murphy SA, Stanek HG, Das SR, Vongpatanasin W (2005). Race and gender differences in C-reactive protein levels. J Am Coll Cardiol.

[30] Lin CTH, Kang L (2016). Adipose extracellular matrix remodelling in obesity and insulin resistance. Biochem Pharmacol.

[31] Nagase H, Visse R, Murphy G (2006). Structure and function of matrix metalloproteinases and TIMPs. Cardiovasc Res.

[32] Alexander CM, Selvarajan S, Mudgett J, Werb Z (2001). Stromelysin-1 regulates adipogenesis during mammary gland involution. J Cell Biol.

[33] Gao D, Bing C (2011). Macrophage-induced expression and release of matrix metalloproteinase 1 and 3 by human preadipocytes is mediated by IL-1β via activation of MAPK signaling. J Cell Physiol.

[34] Maquoi E, Demeulemeester D, Vörös G, Collen D, Lijnen HR (2003). Enhanced nutritionally induced adipose tissue development in mice with stromelysin-1 gene inactivation. Thromb Haemost.

[35] Wu Y, Lee MJ, Ido Y, Fried SK (2017). High-fat diet-induced obesity regulates MMP3 to modulate depot- and sex-dependent adipose expansion in C57BL/6J mice. Am J Physiol Endocrinol Metab.

[36] Alexander CM, Selvarajan S, Mudgett J, Werb Z (2001). Stromelysin-1 regulates adipogenesis during mammary gland involution. J Cell Biol.

[37] Gao D, Bing C (2011). Macrophage-induced expression and release of matrix metalloproteinase 1 and 3 by human preadipocytes is mediated by IL-1β via activation of MAPK signaling. J Cell Physiol.

[38] Maquoi E, Demeulemeester D, Vörös G, Collen D, Lijnen HR (2003). Enhanced nutritionally induced adipose tissue development in mice with stromelysin-1 gene inactivation. Thromb Haemost.

[39] Wu Y, Lee MJ, Ido Y, Fried SK (2017). High-fat diet-induced obesity regulates MMP3 to modulate depot- and sex-dependent adipose expansion in C57BL/6J mice. Am J Physiol Endocrinol Metab.

