# Supplementary material for: Effect of a diet based on the dietary guidelines for americans on inflammation markers in women at risk for cardiometabolic disease: results of a randomized, controlled trial
Source: BMC Nutr. 2022 Dec 27;8:157. doi: 10.1186/s40795-022-00647-z (PMC9793666; doi:10.1186/s40795-022-00647-z)
Supplement: Supplementary file 1 — Additional file 1. [file 40795_2022_647_MOESM1_ESM.docx]

**
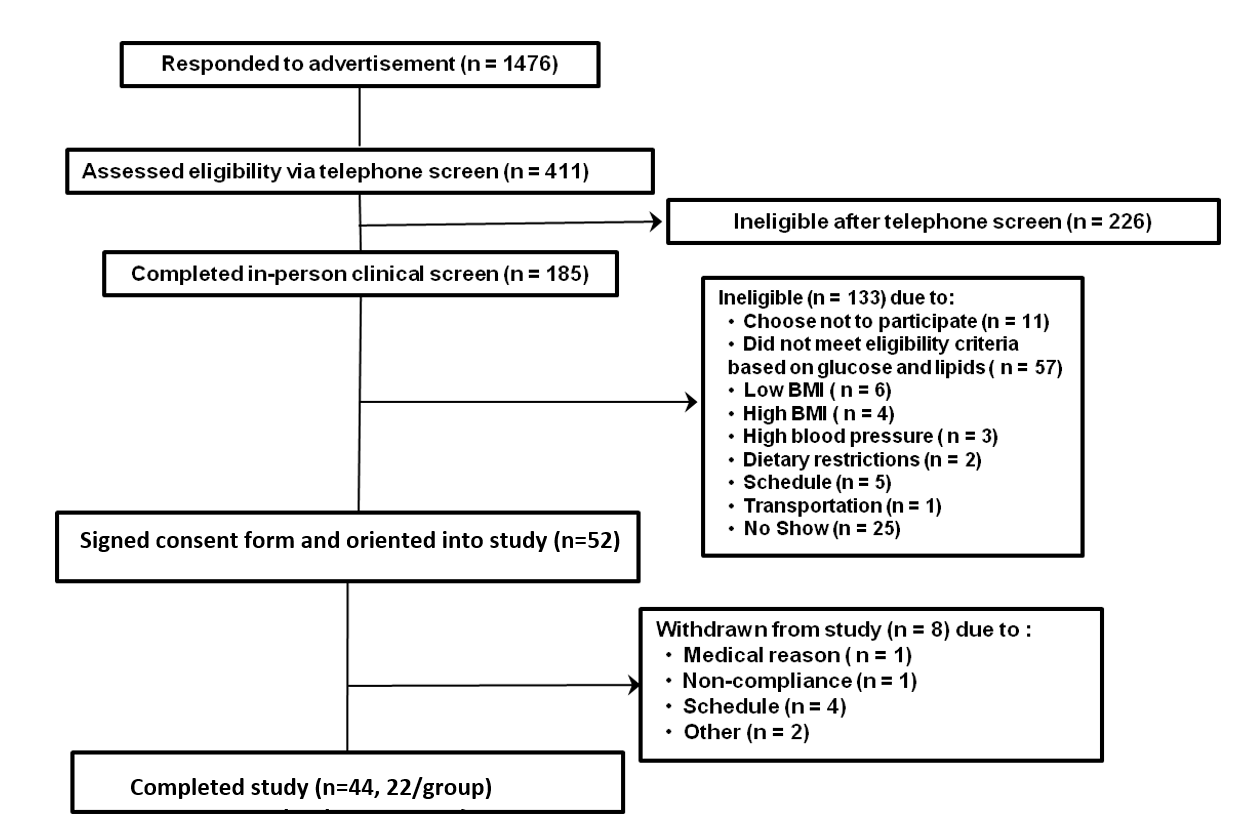
**

**Supplemental Figure 1:** CONSORT diagram representing the volunteers who were screened, consented, and completed the study. CONSORT, Consolidated Standards. In the current study completed study volunteers were used in analysis.

**
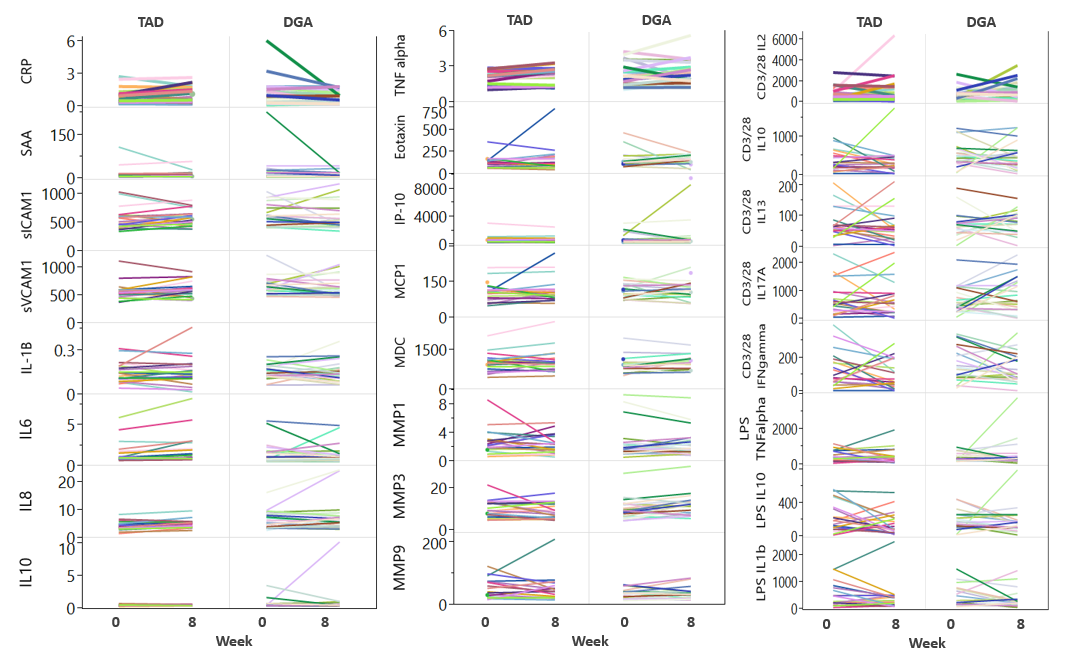
**

**Supplemental figure 2:** Each participant (represented by several colors) at weeks 0 and 8 immunological markers overlayed to show within group differences.

**Supplemental Table 1**: Characteristics of study volunteers (completers) measured at screening, by diet group^1^

| **Anthropometrics** | **TAD (n = 22)** | | **DGA (n = 22)** | |
| --- | --- | --- | --- | --- |
|  | **Mean ± SD** | **Range**  **(Min – Max)** | **Mean ± SD** | **Range**  **(Min – Max)** |
| Age (y) | 47.7 ± 9.6 | 26-64 | 50.7 ± 12.6 | 21-63 |
|  |  |  |  |  |
| Height (cm) | 164.5 ± 7.9 | 152.1 – 179.6 | 165.8 ± 7.0 | 147.2 – 179.0 |
|  |  |  |  |  |
| Weight (kg) | 87.7 ± 13.1 | 65.2 – 115.4 | 88.3 ± 15.6 | 59.9 – 115.4 |
|  |  |  |  |  |
| BMI (kg/m^2^) | 32.5 ± 3.7 | 26.5 – 39.1 | 31.9 ± 4.0 | 25.2 – 38.1 |
|  |  |  |  |  |
| Waist to hip ratio | 0.81 ± 0.08 | 0.6 – 0.9 | 0.81 ± 0.06 | 07 – 0.9 |
|  |  |  |  |  |
| Waist circumference (cm) | 97.0 ± 14.7 | 81.3 – 149.5 | 94.7 ± 9.3 | 78.7 – 111.0 |
| Systolic Blood Pressure (mmHg)  Diastolic Blood Pressure (mmHg) | 119.5 ± 8.7 | 100.7 – 132.8 | 125.3 ± 10.7* | 104.2 – 142.0 |
|  | 72.3 ± 7.9 | 58.7 – 89.0 | 72.2 ± 7.2 | 58.0 – 83.0 |
|  |  |  |  |  |
| **Clinical parameters** |  |  |  |  |
| Fasting glucose (mg/dL) | 89.6 ± 7.2 | 77 – 105 | 92.6 ± 9.7 | 78 – 112 |
|  |  |  |  |  |
| 2-Hour glucose (mg/dL) | 127.2 ± 34.6 | 78 – 188 | 129.9 ± 34.4 | 63 – 199 |
|  |  |  |  |  |
| Fasting insulin (mIU/mL) | 12.7 ± 7.6 | 4.2 – 33.3 | 13.4 ± 9.3 | 4.7 – 35.9 |
|  |  |  |  |  |
| 2-Hour insulin (mIU/mL) | 95.0 ± 83.4 | 4.5 – 366.4 | 86.3 ± 71.9 | 8.4 – 320.4 |
|  |  |  |  |  |
| HOMA-IR | 2.9 ± 1.9 | 0.9 – 8.6 | 3.1 ± 2.3 | 0.9 – 9.1 |
|  |  |  |  |  |
| QUICKI | 0.34 ± 0.03 | 0.28 – 0.39 | 0.34 ± 0.03 | 0.28 – 0.39 |
|  |  |  |  |  |
| Matsuda index | 4.5 ± 3.0 | 0.9 – 11.3 | 4.3 ± 2.6 | 0.7 – 9.6 |
| McAuley index | 10.1 ± 1.4 | 8.2 - 12.7 | 9.9 ± 1.2 | 7.4 - 12.8 |
|  |  |  |  |  |
| HbA1c | 5.6 ± 0.3 | 5.0 – 6.2 | 5.6 ± 0.3 | 5.0 – 6.1 |
|  |  |  |  |  |
| Total cholesterol (mg/dL) | 211.1 ± 25.9 | 149 – 285 | 216.8 ± 39.2 | 154 – 299 |
|  |  |  |  |  |
| LDL-c (mg/dL) | 130.5 ± 20.15 | 90 – 170 | 122.0 ± 30.2 | 70 – 170 |
| HDL-c (mg/dL) | 49.2 ± 11.7 | 27 – 77 | 53.2 ± 10.6 | 33 – 75 |
|  |  |  |  |  |
| Triglycerides (mg/dL) | 143.0 ± 70.7 | 56 – 282 | 151.3 ± 89.8 | 57 – 329 |
| ^1^ Values are means ± SD (min – max); Abbreviations: HOMA-IR, homeostatic model assessment of insulin resistance; QUICKI, qualitative insulin sensitivity check index; HbA1c, glycated hemoglobin; LDL-c, low density lipoprotein cholesterol; HDL-c, high density lipoprotein cholesterol. “*” indicates significantly different from TAD by p ≤ 0.05, by van der Waerdan’s non-parametric test. | | | | |

**Supplemental Table 2:** Mean difference with upper and lower 95% confidence interval of change between week 8 and week 0 in groups DGA and TAD.

| **Parameter** | **TAD** | | | **DGA** | | |
| --- | --- | --- | --- | --- | --- | --- |
|  | **Mean** | **Lower CI** | **Upper CI** | **Mean** | **Lower CI** | **Upper CI** |
| **SAA (mg/L)** | -2.24 | -9.99 | 5.51 | -12.6 | -32.3 | 7.04 |
| **CRP (mg/L)** | 1.69 | -0.56 | 3.94 | -3.71 | -8.75 | 1.33 |
| **sVCAM1 (ng/mL)** | 48.2 | -14.0 | 110 | -12.4 | -103 | 77.9 |
| **sICAM1 (ng/mL)** | 38.1 | -35.6 | 112 | -18.3 | -97.3 | 60.6 |
| **IL-6 (pg/mL)** | 0.427 | 0.126 | 0.73 | -0.10 | -0.69 | 0.48 |
| **IL-1β(pg/mL)** | 0.00392 | -0.0219 | 0.0298 | 0.00249 | -0.0226 | 0.0276 |
| **IL-8 (pg/mL)** | 0.528 | 0.074 | 0.983 | 0.964 | -0.755 | 2.68 |
| **IL-10 (pg/mL)** | 0.00229 | -0.0291 | 0.0337 | 0.311 | -0.667 | 1.29 |
| **TNF-α (pg/mL)** | 0.195 | 0.0725 | 0.318 | 0.131 | -0.2027 | 0.464 |
| **Eotaxin (pg/mL)** | 25.7 | -36.2 | 87.6 | -10.5 | -57.1 | 36.1 |
| **IP-10 (pg/mL)** | -34.5 | -119.0 | 50.0 | 687.0 | -445 | 1819 |
| **MCP1 (pg/mL)** | 2.45 | -21.0 | 25.9 | 2.87 | -26.9 | 32.6 |
| **MDC (pg/mL)** | 28.2 | -110 | 166 | 72.8 | -132 | 278 |
| **MMP-1 (pg/mL)** | -0.283 | -1.021 | 0.455 | 0.00149 | -0.462 | 0.465 |
| **MMP-3 (pg/mL)** | -1.17 | -2.79 | 0.455 | 1.47 | 0.115 | 2.82 |
| **MMP-9 (pg/mL)** | -0.981 | -16.7 | 14.7 | 2.72 | -4.16 | 9.59 |
| **PBMC treatments** | | | | | | |
| **CD328-IL-2 (pg/mL)** | 266 | -269 | 801 | 331 | -114 | 776 |
| **CD328-IL-10 (pg/mL)** | -28.2 | -221 | 165 | -65.2 | -260 | 130 |
| **CD328-IL-13 (pg/mL)** | -9.53 | -37.5 | 18.5 | 6.15 | -17.5 | 29.8 |
| **CD328-IL-17α (pg/mL)** | 3.55 | -267 | 274 | 205 | -68.2 | 479 |
| **CD328-IFN-γ (pg/mL)** | -24.8 | -76.0 | 26.3 | -27.4 | -79.8 | 25.0 |
| **LPS-IL-10 (pg/mL)** | 27.6 | -51.3 | 106 | 22.8 | -63.7 | 109 |
| **LPS-IL-1β (pg/mL)** | 9.8 | -172 | 192 | -38.9 | -213 | 135 |
| **LPS-IL-6 (pg/mL)** | 0.222 | -0.322 | 0.766 | -0.0918 | -0.428 | 0.245 |
| **LPS-TNF-α (pg/mL)** | 90.9 | -110 | 292 | 174 | -202 | 551 |
